# Pruritic eruption in a young woman with fever

**DOI:** 10.1093/skinhd/vzaf039

**Published:** 2025-07-25

**Authors:** Delwyn Zhi Jie Lim, Valencia Long, Lester Juay

**Affiliations:** National Skin Centre, Singapore, Singapore; Division of Dermatology, Department of Medicine, National University Hospital, National University Health System, Singapore, Singapore; Division of Dermatology, Department of Medicine, National University Hospital, National University Health System, Singapore, Singapore

## Abstract

A 29-year-old woman presented with 3 weeks of fever, arthralgia and pruritic rashes on her trunk and limbs. She had no significant medical history and denied occurrence of muscle weakness, dysphagia, mouth ulcers, alopecia and sicca symptoms. Physical examination revealed erythematous, oedematous papules and plaques on the anterior sternum, upper to mid-back, arms, flanks and lateral aspect of the proximal thighs. Antinuclear antibody and anti-double-stranded DNA were negative. Complements, creatine kinase and myositis panel were normal. Her erythrocyte sediment rate and ferritin levels were elevated at 48 mm h^–1^ and 580 µg L^–1^, respectively. Histological examination of the plaque on her upper back revealed epidermal dyskeratosis, with a mixed perivascular infiltrate of neutrophils, lymphocytes and histiocytes within the superficial dermis. There was prominent nuclear dust, red cell extravasation and increased dermal mucin. Direct immunofluorescence was negative. She was eventually diagnosed with persistent pruritic eruption (PPE) of adult-onset Still disease (AOSD) and was treated with oral etoricoxib with resolution of her fever and symptoms. The entity of PPE in AOSD is associated with the striking histological feature of dyskeratotic keratinocytes in the upper one-third of the epidermis. This is in contrast to other conditions such as cutaneous lupus or dermatomyositis, where the dyskeratotic keratinocytes are found in the lower epidermis instead. This case highlights the importance of clinicopathological correlation and recognition of PPEs in AOSD in view of the associated poorer prognosis due to associated conditions such as secondary macrophage activation syndrome.

## Teaser

A 29-year-old woman presented with 3 weeks of fever, arthralgia and pruritic rashes on her trunk and limbs with examination revealing erythematous, oedematous papules and plaques on the anterior sternum, upper to mid-back, arms, flanks and lateral aspect of proximal thighs. Her ferritin levels were raised and a skin biopsy of the plaque on her upper back revealed epidermal dyskeratosis, with a mixed perivascular infiltrate of neutrophils, lymphocytes and histiocytes within the superficial dermis with prominent nuclear dust, red cell extravasation and increased dermal mucin.

What is already known about this topic?

- Adult-onset Still disease (AOSD) is a rare systemic inflammatory disorder characterized by spiking fevers, arthralgias, multiorgan involvement and varied cutaneous manifestations; due to its protean clinical manifestations, diagnosis is often missed or delayed.

What does this study add?

- From a histopathological standpoint, the presence of upper epidermal dyskeratosis with apoptotic keratinocytes and a mixed lymphocyte and neutrophil infiltrate in the superficial dermis has been reported to be fairly specific for pruritic papular eruptions seen in AOSD, which was seen in our patient.
- This case highlights the importance of clinicopathological correlation and recognition of the clinical and pathological features of pruritic papular eruptions in AOSD in view of the associated poorer prognosis.

## Case report

**Figure 1:**
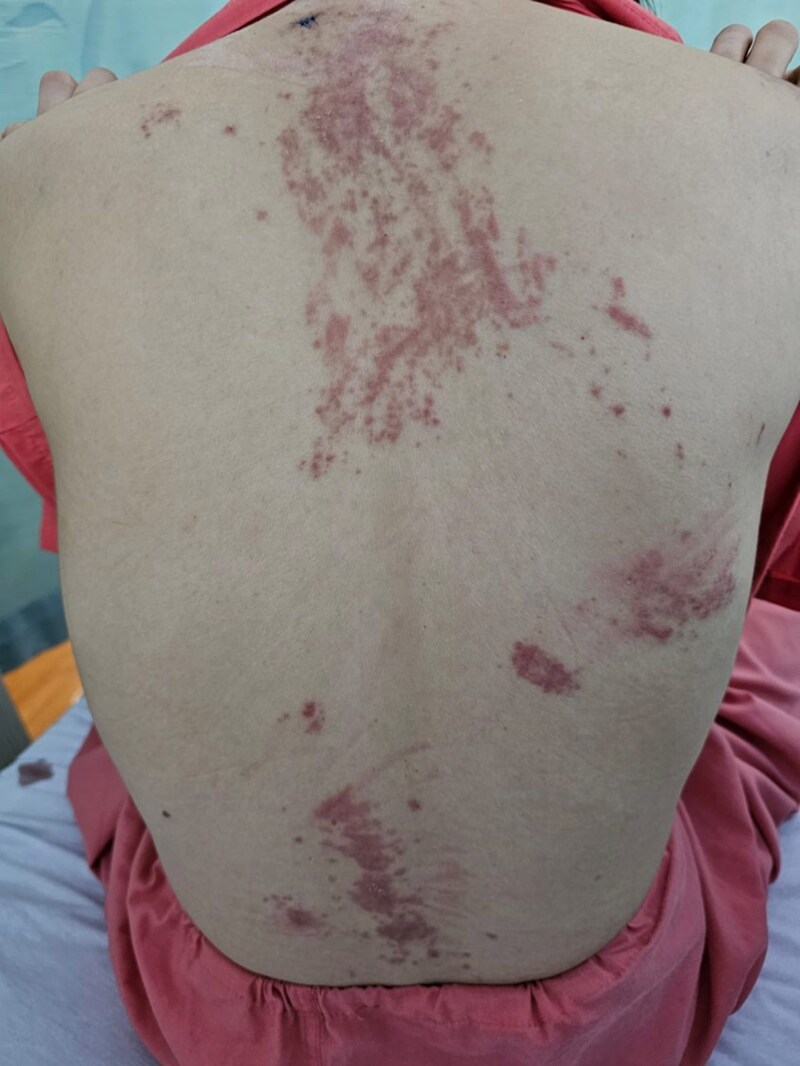
Erythematous, oedematous papules and plaques on the anterior sternum, upper to mid-back.

**Figure 2:**
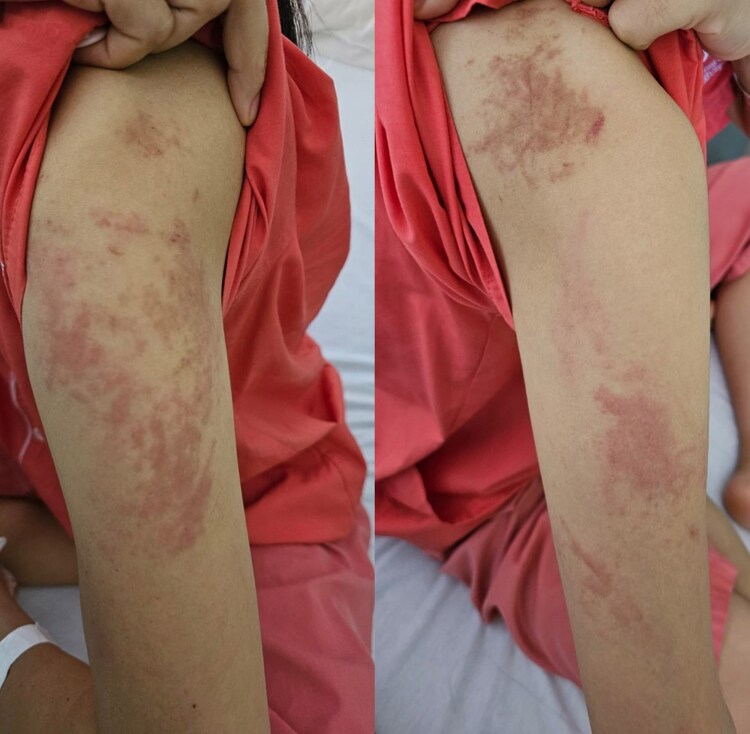
Erythematous, oedematous papules and plaques on the patient’s arms.

A 29-year-old woman presented with a 3-week history of fever, arthralgia and pruritic rashes on her trunk and limbs. She had no significant medical history and denied muscle weakness, dysphagia, mouth ulcers, alopecia and sicca symptoms. Physical examination revealed erythematous, oedematous papules and plaques on the anterior sternum, upper to mid-back (Figure 1), arms (Figure 2), flanks and lateral aspect of the proximal thighs. Gottron’s sign was negative and there was no heliotrope rash. Her palms and soles were uninvolved. There was no lymphadenopathy or hepatosplenomegaly.

Antinuclear antibody (ANA), anti-double-stranded DNA (dsDNA), anti-neutrophilic cytoplasmic antibodies and rheumatoid factor (RF) were negative. Complements were normal. Creatine kinase and aldolase levels were normal, and a myositis panel was negative. Her erythrocyte sedimentation rate (ESR) and ferritin levels were elevated at 48 mm h^–1^ and 580 µg L^–1^, respectively. Computed tomography of her thorax, abdomen and pelvis did not reveal any organomegaly or features of malignancy.

**Figure 3:**
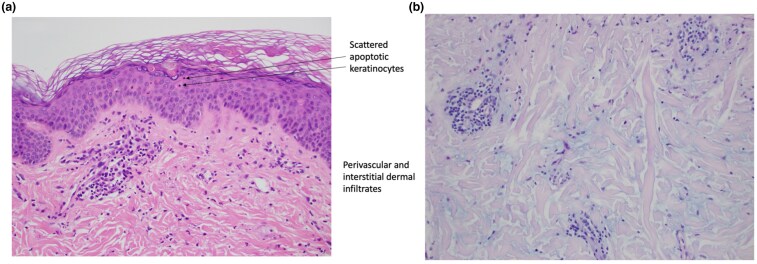
(a) Epidermal dyskeratosis, with a mixed perivascular infiltrate of neutrophils, lymphocytes and histiocytes within the superficial dermis. (b) There was prominent nuclear dust, red cell extravasation and increased dermal mucin.

A skin biopsy was done over the plaque on her upper back and histological examination revealed epidermal dyskeratosis, with a mixed perivascular infiltrate of neutrophils, lymphocytes and histiocytes within the superficial dermis (Figure 3a). There was prominent nuclear dust, red cell extravasation and increased dermal mucin (Figure 3b). There was no fibrinoid necrosis of the capillaries. Direct immunofluorescence was negative.

She was eventually diagnosed with adult-onset Still disease (AOSD) and she fulfilled the Yamaguchi criteria of AOSD of fever, arthralgia, typical rash, leucocytosis and negative tests for ANA and RF. She was treated with oral etoricoxib with resolution of her rashes and symptoms. The patient was not keen to use oral corticosteroids in fear of side effects, nor was she keen on an interleukin-6 inhibitor (tocilizumab) or interleukin-1 receptor antagonist (anakinra) in view of cost. She was subsequently lost to follow-up.

## Discussion

AOSD is a rare systemic inflammatory disorder characterized by spiking fevers, arthralgias, multiorgan involvement and varied cutaneous manifestations. The most common cutaneous manifestation is an evanescent salmon-pink maculopapular eruption that frequently coincides with the febrile phase of the disease. However, other types of cutaneous manifestations have been observed and reported, such as persistent pruritic eruptions (PPEs) – which include urticarial lesions, generalized erythema, prurigo pigmentosa-like eruption and flagellate erythema.^1^ In contrast to the classic evanescent rash, PPEs do not resolve when the fever abates.

It is important to recognize the entity of PPEs as they can mimic cutaneous manifestations of other diseases such as dermatomyositis, bleomycin toxicity and shiitake mushroom intake. Furthermore, the presence of PPEs in AOSD has been associated with the development of a secondary macrophage activation syndrome (MAS), possibly conferring a worse prognosis and higher mortality rates.^2^

As with its clinical features, a broad histological spectrum has been reported in AOSD.^3^ However, the presence of upper epidermal dyskeratosis with apoptotic keratinocytes and a mixed lymphocyte and neutrophil infiltrate in the superficial dermis has been reported to be fairly specific for PPE in AOSD.^4^ The main histological distinguishing feature of PPEs in AOSD is the presence of dyskeratotic keratinocytes in the upper one-third of the epidermis. This is in contrast to other causes of epidermal dyskeratotic keratinocytes such as connective tissue diseases and drug eruptions. Cutaneous lupus erythematosus and dermatomyositis often have dyskeratotic keratinocytes in the lower epidermis instead, while drug eruptions usually present with dyskeratotic keratinocytes throughout all layers of the epidermis.^4^ The diagnosis of AOSD can be challenging and often delayed due to its protean manifestations.

Apart from clinical and histological features, laboratory investigations can also play a role in aiding the clinician to clinch the diagnosis of AOSD. Acute-phase reactants such as ESR, C-reactive protein, leucocyte and ferritin levels are often elevated during flares in AOSD. However, these levels should be taken at the time of flare as they may normalize in between attacks.^5^

It is also important to recognize that AOSD may be associated with the development of MAS, a potentially lethal, severe inflammatory systemic disease that requires urgent treatment. The inflammation is caused by the uncontrolled activation of macrophages and T cells.^6^ While there exist various diagnostic criterions, the distinctive features of MAS are characterized by fever, hepatopathy, hypertriglyceridaemia, hyperferritinaemia, decrease in ESR levels, pancytopenia, coagulopathy and histopathological evidence of haemophagocytosis. Dermatologists should be cognizant of this syndrome for early recognition and accurate treatment for reduction of mortality for these patients.

This patient was eventually diagnosed with PPE of AOSD and also fulfilled the Yamaguchi criteria for diagnosis of AOSD. She had no evidence of MAS. Other conditions such as cutaneous lupus erythematous and dermatomyositis were initially considered but were deemed unlikely diagnoses once the ANA, dsDNA and complement levels returned to normal, and the myositis panel returned negative, respectively. While the diagnosis of AOSD is typically based on the Yamaguchi criteria, pitfalls in the criteria must also be recognized as the criteria focuses on nonpruritic salmon-pink rashes as a cutaneous feature, which may not always be present. The presence of persistent pruritic eruptions should prompt the clinician to consider AOSD as a differential even though it may not be part of the Yamaguchi criteria.^7^ A trial of treatment with oral etoricoxib was also effective in treating her fever, symptoms and rashes, strengthening the case for AOSD. Other potential treatment options include oral corticosteroids, oral methotrexate, interleukin-6 inhibitors (e.g. tocilizumab) or an interleukin-1 receptor antagonist (e.g. anakinra).

This case highlights the importance of clinicopathological correlation and recognition of PPEs in AOSD in view of the possibility of an associated poorer prognosis.

## Data Availability

The data underlying this article will be shared on reasonable request to the corresponding author.
